# Data on thermal sensation, perception and microclimatic parameters in a city with Mediterranean climate

**DOI:** 10.1016/j.dib.2018.12.016

**Published:** 2018-12-11

**Authors:** Areti Tseliou, Ioannis Tsiros, Spyridon Lykoudis

**Affiliations:** a Zayed University, Dubai, UAE; b Agricultural University of Athens, Greece; c Independent Researcher Enargia WG, Greece

## Abstract

This data article presents the data collected through an extensive research work conducted in urban areas in the city of Athens (Greece) during the period 2010–2012. Data concerns 2287 questionnaires and microclimatic data collected through interviews to the visitors of the examined areas with parallel monitoring of the urban microclimatic characteristics. The field surveys carried out occasionally throughout the year covering as much as possible the different seasons under Mediterranean climate conditions. These data are related to the research articles with the titles: Seasonal differences in thermal sensation in the outdoor urban environment of Mediterranean climates–the example of Athens, Greece (Tseliou et al., 2017) and Outdoor thermal sensation in a Mediterranean climate (Athens): The effect of selected microclimatic parameters (Tseliou et al., 2016).

**Specifications table**

**Table:** 

Subject area	*Bioclimatology*
More specific subject area	*Thermal comfort in the urban environment*
Type of data	*Excel file, word file*
How data was acquired	*field surveys were carried out including both microclimatic monitoring with the use of a portable mini weather station (Campbell CR10 data logger) and structured questionnaires related to thermal sensation*
Data format	*Raw, analyzed*
Experimental factors	*2287 interviewees, both genders, all the age groups*
Experimental features	*Use of a portable mini weather station monitoring the urban microclimatic characteristics during the surveys*
Data source location	*Athens (37058*′*46*″ *N 23042*′*58*″ *E), Greece*
Data accessibility	*Data accompany this data article*
Related research article	*Tseliou, A., Tsiros, I. X., &Nikolopoulou, M. (2017) Seasonal differences in thermal sensation in the outdoor urban environment of Mediterranean climates–the example of Athens, Greece. International Journal of Biometeorology, 1–18.*

**Value of the data**.

**These data are valuable for the here under reasons:**

• The data present the microclimatic characteristics of typical urban areas of a Mediterranean city.
• The data can be used to investigate the actual thermal sensation and thermal preferences of the population of a city with Mediterranean climate.
• The data can be used for comparisons with similar data retrieved from field surveys conducted in cities with different microclimatic characteristics.
• Meteorological parameters can be used for the estimation of bioclimatic indices.

## 1 Data

The data presented in this data article include questionnaire data that provide information regarding the thermal sensation and the thermal preferences of 2287 individuals throughout the year [1], and microclimatic variable data collected during surveys using a mini weather station (**excel file, word file**).

## 2 Experimental design, materials, and methods

The described research work consists of three main frameworks as presented here under:

### 2.1 Field surveys

The field surveys were conducted during the months of October, November, March and April consisting the cool months of the year and the months May, June and July consisting of the warm months of the year based on the climatic characteristics of the city.

### 2.2 Questionnaires

The structured questionnaires used for the investigation of human thermal sensation include the following questions along with the code numbers of each question:

• Thermal sensation (ATSV): Very cold (−3), Cold (−2), Slightly cool (−1), Neither cool nor warm (0), Slightly warm (1), hot (2), Very hot (3)
• Sun sensation: Gloomy (−2), Little sun (−1), Pleasant (0), Sunny (1), Too much sun (2)
• Wind sensation: Stale (−2), Little wind (−1), Pleasant (0), Windy (1), Too much wind (2)
• How do you feel at the moment: Comfortable (0), uncomfortable (1).

### 2.3 Microclimatic monitoring

**Fig. 1:**
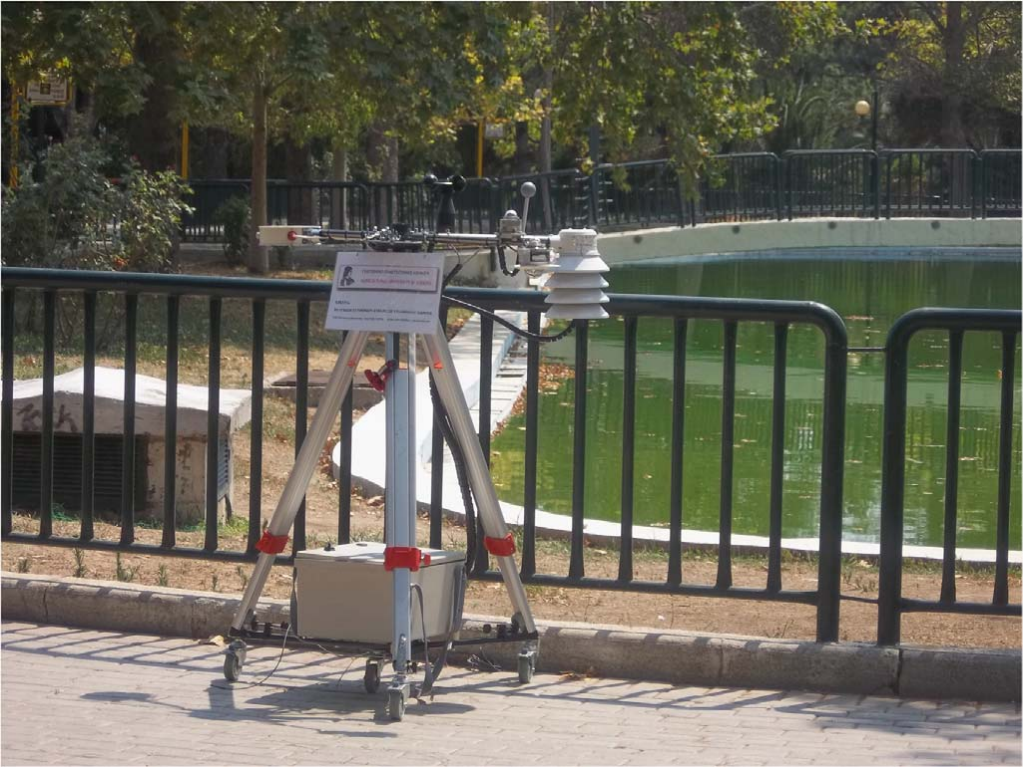
Portable mini weather station.

A portable meteorological station was used to monitor the microclimatic conditions during the field survey [2]. Micrometeorological instruments were positioned at a height of 1.1 m on a wheeled tripod **(**Fig. 1**)**. All instrument readings were stored every 5 min using a Campbell CR10 data logger. For the monitor of air temperature, RHT2-type sensors were used (Delta-T Devices; accuracy ±0.5 °C and ±2%, respectively). Wind speed was measured using a cup anemometer (Vector Instruments Model A100L2; accuracy ±1%, threshold 0.15 m s−1). Solar radiation was measured using a CM7 albedometer that measures the albedo using two pyranometers combined into one instrument (4–6 μV/ (W m^−2^) sensitivity and ±1% error). Globe temperature was measured using a Pt100 sensor inserted into a 38-mm diameter hollow acrylic sphere, painted with flat grey matte black paint.

## References

[1] Tseliou A., Tsiros I.X., Nikolopoulou M. (2017). Seasonal differences in thermal sensation in the outdoor urban environment of Mediterranean climates–the example of Athens, Greece. Int. J. Biometeorol..

[2] Tseliou A., Tsiros I.X., Nikolopoulou M., Papadopoulos G. (2016). Outdoor thermal sensation in a Mediterranean climate (Athens): the effect of selected microclimatic parameters. Arch. Sci. Rev..

