# Supplementary material for: Data on thermal sensation, perception and microclimatic parameters in a city with Mediterranean climate
Source: Data Brief. 2018 Dec 11;22:563–5. doi: 10.1016/j.dib.2018.12.016 (PMC6321971; doi:10.1016/j.dib.2018.12.016)
Supplement: Supplementary file 1 — Supplementary material. [file mmc1.docx]

Dear Editors,

There is no any case of conflict. The data provided concern the authors research and no one else has any right on them.

Thank you,

Areti
